# Allele mining of *TaGRF-2D* gene 5’-UTR in *Triticum aestivum* and *Aegilops tauschii* genotypes

**DOI:** 10.1371/journal.pone.0231704

**Published:** 2020-04-16

**Authors:** Pavel Yu. Kroupin, Anastasiya G. Chernook, Mikhail S. Bazhenov, Gennady I. Karlov, Nikolay P. Goncharov, Nadezhda N. Chikida, Mikhail G. Divashuk

**Affiliations:** 1 Laboratory of Applied Genomics and Crop Breeding, All-Russia Research Institute of Agricultural Biotechnology, Moscow, Russia; 2 Institute of Cytology and Genetics, Siberian Branch of the Russian Academy of Sciences, Novosibirsk, Russia; 3 Federal Research Center Vavilov All-Russian Institute of Plant Genetic Resources, Saint Petersburg, Russia; 4 Centre for Molecular Biotechnology, Russian State Agrarian University–Moscow Timiryazev Agricultural Academy, Moscow, Russia; 5 Kurchatov Genomics Center-ARRIAB, All-Russia Research Institute of Agricultural Biotechnology, Moscow, Russia; Institute of Genetics and Developmental Biology Chinese Academy of Sciences, CHINA

## Abstract

The low diversity of the D-subgenome of bread wheat requires the involvement of new alleles for breeding. In grasses, the allelic state of *Growth Regulating Factor* (*GRF*) gene is correlated with nitrogen uptake. In this study, we characterized the sequence of *TaGRF-2D* and assessed its diversity in bread wheat and goatgrass *Aegilops tauschii* (genome DD). *In silico* analysis was performed for reference sequence searching, primer pairs design and sequence assembly. The gene sequence was obtained using Illumina and Sanger sequencing. The complete sequences of *TaGRF-2D* were obtained for 18 varieties of wheat. The polymorphism in the presence/absence of two GCAGCC repeats in 5’ UTR was revealed and the GRF-2D-SSR marker was developed. Our results showed that the alleles 5’ UTR-250 and 5’ UTR-238 were present in wheat varieties, 5’ UTR-250 was presented in the majority of wheat varieties. In *Ae*. *tauschii* ssp. *strangulata* (likely donor of the D-subgenome of polyploid wheat), most accessions carried the 5’ UTR-250 allele, whilst most *Ae*. *tauschii* ssp. *tauschii* have 5’ UTR-244. The developed GRF-2D-SSR marker can be used to study the genetic diversity of wheat and *Ae*. *tauschii*.

## Introduction

Gene diversity of bread wheat compared to its diploid progenitors was significantly reduced due to domestication bottleneck [1]. In the last century, genetic diversity of wheat was partially lost as a result of the replacement of local landraces by modern elite cultivars [2, 3]. In commercial cultivars, that have been extensively introduced since the Green Revolution, the alleles for the semi-dwarf and photoperiod insensitive (short day length) phenotypes were widely used. The dwarfing alleles express the DELLA protein resistant to gibberellic acid (GA)-mediated proteolysis, which leads to inhibition of cell growth and, as a result, to the dwarf phenotype of plants. At the same time, semi-dwarf and dwarf plants did not lodge even at high doses of nitrogen fertilizers [4, 5]. It was shown in recombinant inbred lines derived from segregating populations that plants homozygous for the dwarfing *Rht* alleles have lower 1000 grain weight compared to tall plants, although, in general, semi-dwarf modern cultivars have higher thousand kernel weight [6–8]. Besides, due to the greater DELLA stability, semi-dwarf isogenic lines have lower nitrogen uptake and are less responsive to higher doses of nitrogen fertilizers than plants with the wild-type *Rht-B1a* allele [9, 10]. Low nitrogen uptake by the plant (N utilization efficiency (NUtE: the amount of grain produced per unit of N uptake) leads to serious environmental problems: reduced grain quality and environmental pollution by fertilizers [11–13]. Thus, the next important step in wheat breeding with a growing people population under conditions of limited resources, including the availability of nitrogen fertilizers, is the improvement of nitrogen uptake with increased 1000 grain weight in the dwarf and semi-dwarf plants.

The process of nitrogen uptake is a complex system of physiological pathways, including many biochemical reactions regulated by multiple genes. *GRF4* is a transcription factor (TF) of many nitrogen metabolism genes in plants. The protein synthesized as a result of *GRF4* expression provides the nitrogen and carbon uptake, as well as homeostatic coordination of nitrogen metabolism in plants. In rice, the *GRF4*^*ngr2*^ allele was identified, which differs from other alleles by nucleotide substitutions in the promoter and resistance to cleavage by miR396. As a result of the interaction of the *GRF4*^*ngr*^ product with other TFs the transcription of nitrogen metabolism genes is activated and an increase in the grain size is observed [10, 14]. It has been shown that in dwarf rice plants the activity of glutamate synthase and nitrate reductase is less than that of tall plants; after the *GRF4ngr2* allele introgression into the dwarf plants genome, the level of activity returned to the level of tall-growing plants. At the level of AMT1.1, GS1.2, GS2, NADH-GOGAT2 nitrogen metabolism proteins gene transcription, the *GRF4ngr2* introgression into the genome of dwarf plants led to the fact that their expression of these genes even exceeded that of tall plants. The mechanism of the expression activation of these genes is as follows. Due to the GRF4 factor with GRF-interacting factor 1 (GIF1) protein interaction, the former more effectively binds to GCGG-containing promoters of the AMT1.1 and GS1.2 genes, thereby activating their expression [10].

Dwarf and semi-dwarf wheat plants carrying the alleles of *Rht* genes conferring dwarf phenotypes have lower protein content than plants of the wild-type genotype [15–17]. Plants of bread wheat (*Triticum aestivum* L.) having the *Rht-B1b* allele and genetically modified with the *GRF4ngr2* allele from rice showed a higher rate of nitrate ion uptake, an increased diameter and wall thickness of a culm (while keeping its dwarfism), with an increased number of grains per spike compared to the initial wheat plant [14]. The QTL in macaroni or durum wheat (*T*. *durum* Desf.), associated with 1000 grain weight, was colocalized with the *OsGRF4* homolog gene on 6A chromosome. The *GRF4-Az* allele was identified, the presence of which is associated with an increased 1000 grain weight in durum wheat compared with other alleles of this gene [18]. Haplotypes that increase the nitrogen uptake in both rice and durum wheat are very rare in the germplasm collections of these crops.

The genome of bread wheat as an allopolyploid includes three subgenomes from various ancestral species, B-, A- and D-genomes. It is suggested that the first hybridization occurred between 0.2 and 1.3 million years ago between *T*. *urartu* Thum. ex Gandil. (genome AA) and *Aegilops speltoides* Taush. (genome SS), resulting in the wild tetraploid wheat emmer speication *T*. *dicoccoides* Körn. ex Aschers. et Graebn.) Schweinf. (= syn. *Triticum turgidum* ssp. *dicoccoides* (Körn. ex Aschers. et Graebn.) Thell.) (BBAA genome). About 8–10 thousand years ago, *Ae*. *tauschii* Coss. (= syn. *Ae*. *squarrosa* L.) with D-genome hybridized with a tetraploid wheat gave rise to the hexaploid [19, 20]. Each time when a new subgenome was included in the wheat polyploid, as a result of the bottleneck effect only a small percentage of the entire variety of genotypes of one or another species participated in the process of wheat polyploidization [21]. The D-subgenome was the last to be included in allohexaploid, thus it is the youngest among all subgenomes, which has the minimum genetic diversity compared to subgenomes A and B due to the bottleneck of polyploidization [22–25]. It is noted that 1D and 2D chromosomes show the greatest diversity among D-genome chromosomes [26]. At the same time the D-subgenome incorporation made wheat a staple food primarily due to the presence of genes for storage protein (high and low molecular weight glutenins, puroindolines and storage proteins activators, SPA), which provide dough elasticity and high bread making quality [27–30]. In addition, it is chromosome 2D where the genes for adaptability to the environment, such as the *Ppd-D1* gene for the response to photoperiod, *Rht-8c* for dwarfism, etc. are located [31–35]. Moreover, chromosome 2D harbors the FRIZZY PANICLE gene responsible for the number of spikelets formation (among homologues of most importance) [36], *C* gene, which determines the compact spike [37], *Iw2* gene, which controls the absence of glaucousness on vegetative and generative organs of plant [38,39], *Pis1* gene, which controls a multi-gynoecium (synonym: three pistils) [40], QTLs of 1000 grain weight [41] (*D1* and *D4* conferring hybrid dwarfism [42, 43] and other genes.

Clustering of wheat varieties into two subgroups (South and East European varieties and modern West and North European varieties), carried out using a large number of PCR markers, was largely due to selection for the *Rht8* locus located on 2D chromosome [44]. It was demonstrated that the diversity of *Ae*. *tauschii* significantly exceeds the diversity of the polyploid wheat D-genome [26, 45]. One of the modern trends in the wide hybridization of wheat over the past half century is an increase in the diversity of bread wheat due to the bread wheat resynthesis using *Ae*. *tauschii* germplasm [46–48]. In addition, the majority of gene introgressions of agronomically important traits from wild related species occurred in the chromosomes of the D-genome and the presence of D chromosomes increases the success of distant interspecific and intergeneric introgressions [49–55]. To increase the efficiency of introgressions from the D-genome, an intensive study of its molecular genetic structure using the NGS approaches is carried out in order to map and develop markers for genes of agronomical important traits [33, 56].

The aim of our work was sequencing the *TaGRF-2D* gene (Ta = *Triticum aestivum)* in bread wheat and *Ae*. *tauschii*, the D-subgenome donor, screening for a nucleotide polymorphism of *TaGRF-2D* in those species and the development of molecular marker for the identification of *TaGRF-2D* allelic variants.

## Materials and methods

### Plant material

The germplasm of 79 varieties of bread wheat (S1 Table) and 37 accessions of *Ae*. *tauschii* of various geographical origin (S2 Table) was kindly provided by the member of Russian Academy of Sciences Prof. L.A. Bespalova (Department of Breeding and Seed Production of Wheat and Triticale, National Center of Grain named after P.P. Lukyanenko, Krasnodar), Dr. Oleg G. Semenov (Department of Technosphere Safety, Agrarian-Technological Institute, RUDN University, Moscow), and Dr. E.D. Badaeva (Laboratory of Genetic identification of plants, Vavilov Institute of General Genetics, Russian Academy of Sciences, Moscow). The *Ae*. *tauschii* accessions partially were provided by the Federal Research Center Vavilov All-Russian Institute of Plant Genetic Resources (VIR, Saint-Petersburg) as a part of the VIR genebank. Grains of 20 breeding lines of winter bread wheat (S5 Table) were kindly provided by Dr. V.N. Igonin (Lisitsyn Field Experimental Station, Russian State Agrarian University–Moscow Timiryazev Agricultural Academy). Grains of each accession were obtained from wheat plants that were grown in 2019 year in Lisitsyn Field Experimental Station (55°50'18.2"N 37°33'13.0"E) under following conditions: presowing treatment of seeds was performed using Maxim fungicide (Syngenta, Basel, Switzerland); rate of sowing was 5 million of germinable seeds per ha in three randomized replicates of 10 m^2^ plot with 14 cm interrow spacing, mustard for seeds as a forecrop; the fertilizers were applied in autumn as basic presowing fertilizer (N_32_P_32_K_32_), in spring (N_75_) and at booting stage (N_35_); the fields were treated with herbicide Alister Grand (Bayer, Leverkusen, Germany) and Amystar Extra (Syngenta, Basel, Switzerland).

### SSR analysis

Genomic DNA was extracted from leaves using a CTAB method [57]. The BLAST-search in the wheat genome assembly IWGSC RefSeq v1.0 using rice OsGrf3 (GenBank BK004858.1) as a query, among the resulting sequences of the D-subgenome, in the first instance (with maximum score and the least e-value) gives the TraesCS2D01G435200 gene, as well as TraesCS2A01G435100 and TraesCS2B01G458400 genes on the homoeologous group-2 chromosomes.

The microsatellite markers located next to *TaGRF-2D* gene were selected based on the annotation of the wheat genome assembly IWGSC RefSeq v1.0 using the genome browser. The markers that were reported to have several loci in wheat genome, were discarded. The microsatellite locus CFD233 (PCR marker primers: F 5'GAATTTTTGGTGGCCTGTGT 3'; R 5'ATCACTGCACCGACTTTTGG 3') was selected as the nearest for the TraesCS2D01G435200 on the 2D chromosome, with a distance of 14 949 152 base pairs (bp). In addition, we used the *Xgwm261* microsatellite marker linked with *Rht-8* gene (PCR primers: F 5' CTCCCTGTACGCCTAAGGC 3'; R 5' CTCGCGCTACTAGCCATTG 3').

PCR was performed in a 25 μL reaction volume, containing 70 mM Tris–HCl buffer (pH 8.6), 16.6 mM (NH_4_)_2_SO_4_, 2.5 mM MgCl_2_, 0.2 mM of each dNTP, 10% v/v dimethyl sulfoxide, 0.3 μM forward and reverse primers (Sintol Ltd., Moscow, Russia), 1.0 U of Colored Taq-polymerase (Sileks Ltd., Moscow, Russia) and 100 ng of template DNA. The PCR conditions were as follows: (1) 95°C for 5 min, (2) 35 cycles of 95°C for 30 sec, 60°C for 30 sec, 72°C for 60 sec; and (3) final extension step of 72°C for 10 min. The PCR products were separated in a 1.5% agarose gel in TBE buffer using GeneRuler 100 bp DNA Ladder (Thermo Fisher Scientific, Waltham, Massachusetts, USA) as a molecular weight marker, and stained with ethidium bromide for subsequent visualization in Gel Doc XR+ (Bio-Rad Laboratories, Inc., Hercules, California, USA).

### *TaGRF-2D* sequencing

The sequence of the *TaGRF-2D* (TraesCS2D01G435200) gene was obtained from the wheat genome assembly IWGSC RefSeq v1.0 using the genome browser. The sequence of the gene was divided into seven overlapping regions with sizes varying from 643 to 1342 bp; for each region the forward and reverse specific PCR primers were designed (S3 Table). The PCR products not only covered the total sequence of the gene, but also 1000 bp of the proximal promotor region (S3 Fig). The primers were designed using the PrimerBLAST NCBI. The check for primer specificity was performed in GeneDoc v2.7 by searching their sequences in the alignment of the three homoeologous genes of the A, B, and D wheat subgenomes.

Finally, the primers designed for amplification of the *TaGRF-2D* gene sequences were tested by the PCR with the DNA of Grom and Altigo varieties. The PCR mixture content for amplification and subsequent sequencing are shown in S3 Table. PCR conditions were as follows: (1) 95°C for 10 min, (2) 45 cycles of 95°C for 30 sec, T for 30 sec, 72°C for 4 min; and (3) final extension step of 72°C for 10 min, where T is annealing temperature shown in S3 Table.

The amplified fragments of *TaGRF-2D* gene were obtained from 18 wheat varieties. For each variety, agarose gel electrophoresis was performed to check if the target fragment is the only amplicon in the tube and if its size is close to the expected one (S4 Fig). The gene fragments obtained from the same wheat variety that possessed satisfactory quality and quantity, were mixed in a single tube and submitted for NGS sequencing. Illumina sequencing was ordered in “Genomed, Ltd.” (Moscow). The DNA libraries were prepared using Swift 2S™ Turbo DNA Library Kits. In the process of library preparation, the content of each tube, corresponding to a single definite wheat variety, was labelled with individual DNA barcode. The sequencing was performed on MiSeq system. After de-barcoding, the results were obtained for each submitted test-tube separately as two files of short paired-end reads. Further, the total sequences of the gene for each wheat variety was reconstructed from the NGS data using the undermentioned algorithm.

To be sure that the revealed difference in the microsatellite length is not an artifact of the gene assembly algorithm, we further sequenced the DNA fragment amplified with the primers GRF-2D-2F/R using a Sanger dideoxy sequencing method on a 3130xl Genetic Analyzer («Applied Biosystems», USA).

### *TaGRF-2D* sequence assembly

The quality of sequencing was assessed using FastQC software. In general, the quality of the obtained reads was sufficient for further analysis. In order to reveal larger insertions or deletions, that could not be detected by standard base-calling programs, first, the NGS data were used for de-novo sequence assembling, and then, the resulting sequences were used for mapping of the reads and detection of smaller polymorphisms, that could present in heterozygous state. First, the contigs were assembled from the paired-end reads using the SPAdes 3.13.0 software [58]. Further, the same reads were mapped on the contigs using the SNAP program [59]. Detection of small polymorphisms (SNPs and indels) was implemented using Freebayes software [60]. The detected polymorphisms were introduced into sequences of contigs using BCFtools (https://github.com/samtools/bcftools). The contigs were assembled into scaffolds with an assistance of the reference sequence of a gene and the ABACAS2 software (https://github.com/sanger-pathogens/ABACAS2). Finishing of the scaffold assembling was performed manually in the GeneDoc v2.7 program using alignment against the reference sequence of a gene [61].

The search for the transcription factors binding sites in the 5’ UTR of a gene was performed using the PlantRegMap online resource (http://plantregmap.cbi.pku.edu.cn/binding_site_prediction.php) for *Triticum aestivum* species. Translation regulatory motifs in the 5ʹ UTR were predicted using UTRSite web service (http://utrsite.ba.itb.cnr.it/). The 2D-images of the hypothetical RNA secondary structure in the 5’ UTR were obtained using the ViennaRNA web service (http://rna.tbi.univie.ac.at/forna/).

### GRF-2D-SSR marker

**Fig 1:**
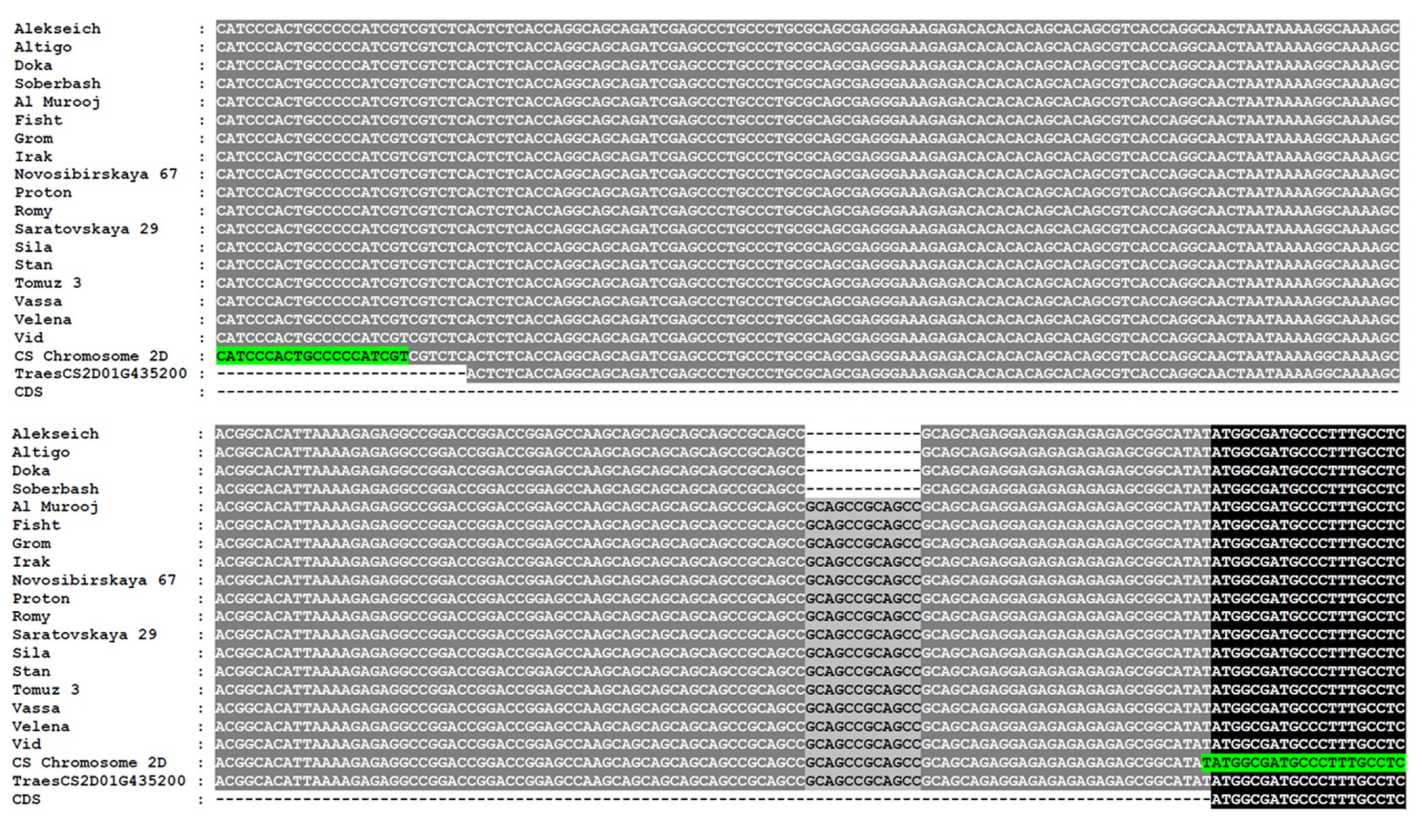
Polymorphism in 5’ UTR of *TaGRF-2D* in 18 bread wheat varieties. Primers for the GRF-2D-SSR marker are highlighted in green. CDS, coding sequence.

The primers for the detection of indel in 5’ UTR of *TaGRF-2D* were designed using the PrimerBLAST NCBI (Fig 1, highlighted in green). The PCR mixture content and annealing temperature for the GRF-2D-SSR marker are shown in S3 Table; PCR conditions are the same as for the primers for the overlapping regions of *TaGRF-2D*. The size of the PCR products amplified from GRF-2D-SSR were measured using fragment analysis in a Genetic Analyzer ABI-3130XL (Applied Biosystems, Foster City, California, USA).

### Allelic state of *Rht* and *Ppd*

The allelic state of *Rht-B1* was identified using PCR markers for *Rht-B1a*/*Rht-B1b* [62] and *Rht-B1a*/*Rht-B1e* [63]; the allelic state of *Ppd-D1* was detected using PCR markers for *Ppd-D1a*/*Ppd-D1b* [64]. PCR conditions were as recommended by the authors, PCR products were separated by electrophoresis as described above.

### Grain phenotyping

Grain length, grain width and thousand grain weight were measured for 20 breeding lines of bread wheat (provided by Dr. V.N. Igonin). Not less than 1500 grains were analyzed for each accession with the use of a Seed Counter application (https://play.google.com/store/apps/details?id=org.wheatdb.seedcounter). Association between the allelic state of *TaGRF-2D*, *Rht-B1*, *Rht-D1*, *Ppd-D1* and the grain parameters was found using one-way ANOVA, the significance of differences was estimated at the level of significance of p < 0.05 using Statistica 12.0 software (StatSoft, Inc., Tulsa, Oklahoma, USA).

## Results

### The diversity of chromosome 2D in the bread wheat varieties

Since the gene we are looking for is located on 2D chromosome, we were tasked to assess the diversity of 79 varieties on this chromosome using *Cfd233* and *Xgwm261* SSR markers (S1 and S2 Figs). As a result of their allelic diversity analysis, we selected 18 varieties which are the most polymorphic for these two SSR markers: Grom, Altigo, Alekseich, Doka, Soberbash, Al-Murooj, Fisht, Iraq, Novosibirskaya 67, Proton, Romy, Saratovskaya 29, Sila, Stan, Tomuz 3, Vassa, Velena, and Vid. These varieties were distinguished not only by allelic diversity, but also by various origin. This group of varieties, as the most diverse according to 2D chromosome, was subsequently used to search for and sequencing the *TaGRF-2D* gene.

### Gene sequencing and sequence analysis

As a result of bioinformatic analysis, we obtained the TraesCS2D01G435200 sequence, the most homologous to the *OsGrf3* sequence of rice. We divided this sequence into 6 fragments; primers were selected for each of them (S3 Table and S3 Fig). The resulting primers in PCR gave fragments corresponding to the expected ones (S4 Fig). Each of the obtained PCR of products was sequenced and, using bioinformatic technologies, a complete gene sequence identical to the TraesCS2D01G435200 sequence was assembled (S3 Fig). The *TaGRF-2D* gene sequence was highly conservative among the studied wheat varieties. A polymorphism in the studied group of varieties was detected by the number of GCAGCC repeats in the 5ʹ-untranslated gene region in positions -42…-31. This motif was repeated twice in the bread wheat varieties Alekseich, Altigo, Doka, and Soberbash (total gene length from start to stop codon 3883 bp) and four times in other varieties (total gene length 3871 bp). Thus, the polymorphism between these varieties is an indel of 12 bp in size (Fig 1). The alternative variants of the *TaGRF-2D* sequences were submitted to GenBank (MT023338 and MT023339).

In order to make sure that microsatellite repeat polymorphism is not an artefact of contiging, we performed Sanger sequencing of all 18 varieties that confirmed the presence of indel of 12 bp in size. The rest of the gene and its promoter were identical in all 18 varieties of wheat. (Figs 1 and S3).

### The marker for the polymorphism in *TaGRF-2D*

**Fig 2:**
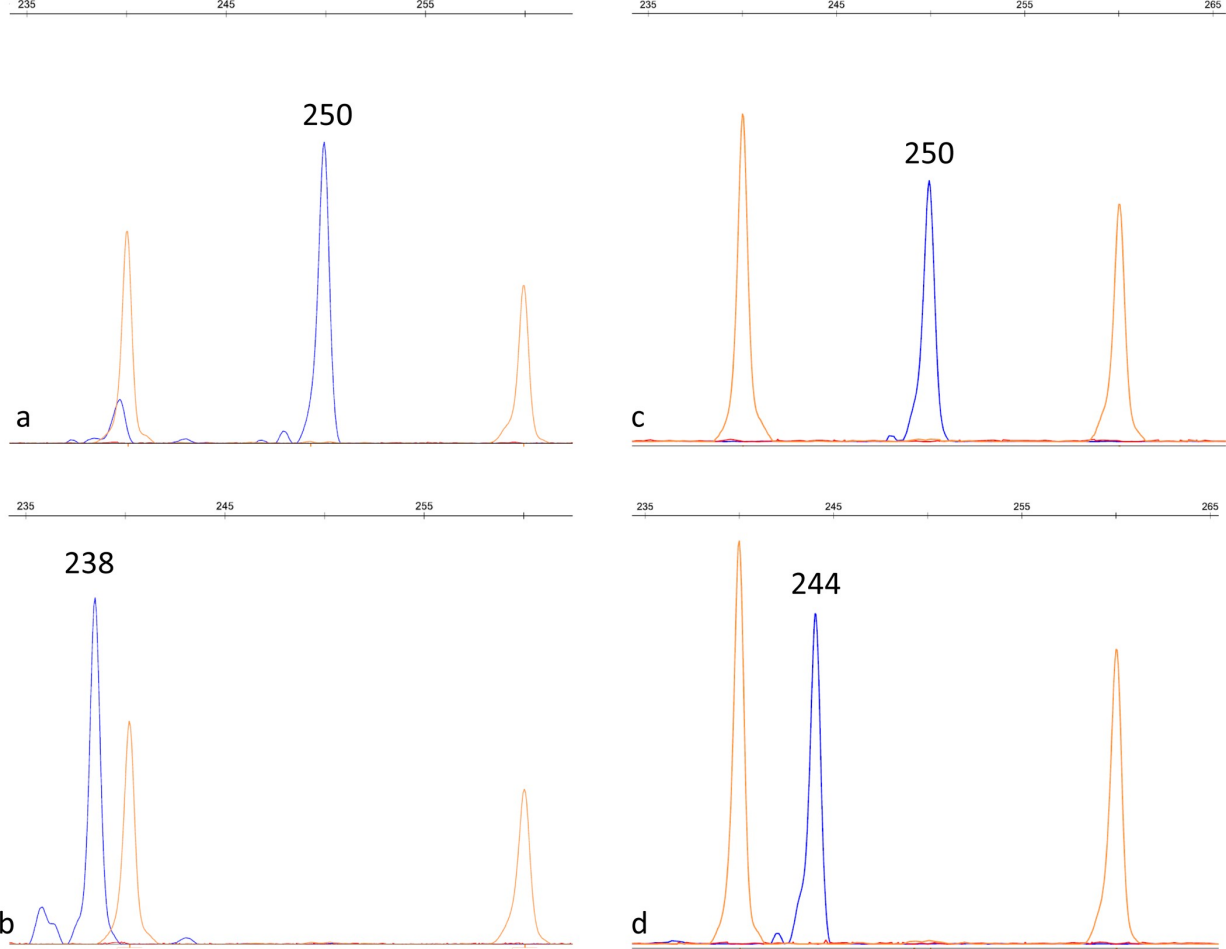
Polymorphism in the GRF-2D-SSR marker. Fragment analysis of the GRF-2D-SSR amplification products in bread wheat (250 bp–a, 238 bp–b) and *Aegilops tauschii* (250 bp–c, 244 bp–d).

The only polymorphic region of *TaGRF-2D* among 18 analyzed wheat varieties was the 5’-untranslated region (5’ UTR). We developed a GRF-2D-SSR marker by selecting primers that detect the detected insertion/deletion in the *TaGRF-2D* gene (S3 Table and Fig 1). As a result of verification of the produced GRF-2D-SSR marker in 18 wheat varieties, we showed that the 238 bp fragment is amplified in the Alekseich, Altigo, Doka and Soberbash varieties, the 250 bp fragment in the other varieties, which is consistent with the sequencing data (Fig 2A and 2B).

Since the developed GRF-2D-SSR marker accurately identified the 12-bp insertion/deletion, we used this marker to screen the entire 79 varieties of bread wheat. Among analyzed varieties, only two types of fragments were revealed, 238 bp in 26 varieties, 250 bp in 53 varieties (S1 Table). The marker we developed was used to screen the *Ae*. *tauschii* germplasm collection. As a result, two types of fragments were revealed, a 244 bp fragment was detected in 20 *Ae*. *tauschii* ssp. *tauschii*, one *Ae*. *tauschii* ssp. *tauschii* var. *meyeri*, one *Ae*. *tauschii* ssp. *tauschii* var. *anathera*, two *Ae*. *tauschii* ssp. *tauschii* var. *typica* and three *Ae*. *tauschii* ssp. *strangulata* accessions; a 250 bp fragment was detected in three *Ae*. *tauschii* ssp. *tauschii*, one *Ae*. *tauschii* ssp. *tauschii* var. *typica* and six *Ae*. *tauschii* ssp. *strangulata* accessions (S2 Table). The identified 5’ UTR allelic variants were designated as 5’ UTR-238, 5’ UTR-244 and 5’ UTR-250.

### Association between the allelic state of *TaGRF-2D*, *Rht* and *Ppd* and grain parameters in the studied germplasm

In order to find out if the allelic state of *Ppd-D1* and *TaGRF-2D*, that are colocalized at chromosome 2D, are associated, we identified the allelic state of *Ppd-D1* in the studied 79-variety wheat germplasm. As a result, we revealed, that 7 varieties carry *Ppd-D1b* allele for photoperiodic sensitivity while the majority of them, 70 varieties, carry *Ppd-D1a* allele for photoperiodic insensitivity. No association between the polymorphism in *TaGRF-2D* and *Ppd-D1* was found that means that their alleles are distributed among the germplasm independently.

We studied 18 varieties, for which a full-length *TaGRF-2D* sequence was obtained, for the presence of the dwarfing alleles *Rht-B1* and *Rht-D1* (partially obtained here using the molecular markers, partially was taken from Divashuk et al. (2012) [65], S4 Table). As a result of the search for *Rht* dwarfing alleles (*Rht-B1b*, *Rht-B1e*, and *Rht-D1b*), *Rht-B1b* and *Rht-B1e* were found in ten and four varieties, respectively; two cultivars carry *Rht-D1b* and two carry the neutral wild-type alleles. No association between the allelic state of *TaGRF-2D* and *Rht* genes was revealed in the studied set of 18 varieties.

We estimated the association between the allelic state *TaGRF-2D* and grain weight and size in a set of 20 bread winter wheat breeding lines grown under the same conditions (Lisitsyn Field Station, RSAU-MTAA, S5 Table). Additionally, we identified the allelic state of *Rht-B1*, *Rht-D1*, and *Ppd-D1* in these lines. No association between the allelic state of *TaGRF-2D*, *Rht* and *Ppd* was revealed in the studied set of 20 breeding lines. We found that the lines with 5’ UTR-238 had higher values of thousand grain weight (p = 0.029), grain length (p = 0.022), and grain width (not significant and p < 0.05) in comparison to the lines with 5’ UTR-250 (S6 Table). Additionally, we found that the breeding lines carrying any of the dwarfing alleles (*Rht-B1b*, *Rht-B1e* or *Rht-D1b*) have on average smaller thousand grain weight than those with neutral wild-type alleles (*Rht-B1a* or *Rht-D1a*) (p = 0.048, S6 Table).

### Prediction of transcription and translation binding sites

We identified the miRNA396 binding site, but no polymorphism has been identified. The analysis of potential transcription factor (TF) binding sites revealed 21 TFs that could hypothetically bind to the studied 5’ UTR (S5 Fig). We compared the possibility of their binding to three alleles we identified: 5’ UTR-238, 5’ UTR-244 and 5’ UTR-250. Some of the TFs showed polymorphism in their ability to bind to different allelic variants of 5’ UTR, as well as in the number of potential binding sites. Six transcription factors that could hypothetically bind only to 5’ UTR-244 and 5’ UTR-250 belong to the ERF (Ethylene Response Factors) family and are associated with the resistance to stress and preharvest sprouting, one to the LBD (Lateral Organ Boundaries Domain) family and is associated with flower development. The analysis of potential translation binding sites showed the potential for binding the translation factors to this site (S7 Table). The analysis of the secondary structure of a hypothetical RNA molecule showed that the RNA transcribed from 5' UTR-238, 5' UTR-244 and 5' UTR-250 differs from each other, while the stem loop structures formed by 5' UTR-244 and 5' UTR -250 are more similar to each other compared to the structure formed by 5' UTR-238 (S6 Fig).

## Discussion

Molecular markers are widely used for the assessment of the genetic diversity of bread wheat and its related species on the genomic level [2, 3, 23, 26] as well for the estimation of allelic variation of the particular genes in collections [29, 34, 44, 65]. The information of allele distribution and genetic diversity is of great importance for the development of marker-assisted wheat breeding strategy. In this study, the GRF-2D-SSR marker was developed base on the polymorphism of the microsatellite motif in the 5’-untranslated region of the *TaGRF-2D* gene of bread wheat. Simple sequence repeats (SSRs) in eukaryotes, including plants, are more often found in UTR than in other transcribed regions, where they serve as the binding sites for the transcription regulators [66,67]. The GCAGCC repeating revealed in the present study in *TaGRF-2D* is located downstream the promoter and hence may be a binding site for TFs. The presence of SSRs increases the UTR variability due to a change in their copy number due to slipped strand mispairing (slippage) during DNA replication or unequal crossing-over upon recombination [67,68]. The 5’ UTR polymorphisms in bread wheat, including repeating sequences, may be associated with phenotypic gene expression [69–72]. Hexanucleotide SSRs have been found to be one the most widespread microsatellites in wheat and can play a functional role [73,74].

The polymorphic fragment revealed in the present study is located between the promoter and the start codon and, therefore, can participate in the binding of TFs at transcription in DNA and/or translation factors at translation in RNA [75]. Indeed, *in silico* analysis showed that the transcription factors can bind to the polymorphic region. We found that the size of a microsatellite in the 5’ UTR hypothetically affects which transcription factors will bind to, as well as the potential number of binding sites: with increasing repetitions, the number of possible TFs and the number of possible binding sites increase. Six TFs that would only bind to the 5’ UTR-244 and 5’ UTR-250 and would not bind to the 5’ UTR-238 belong to the ERF family and primarily bind to GCC boxes. Godoy et al. (2011) revealed in the *Arabidopsis thaliana* that GCAGCC occurred as a result of a mutation in the GCC box and refers to the GCC-like boxes [76]. la Rosa et al. (2014) demonstrated that the RAP2.3 transcription factor (a group of response factors for ethylene, developmental proteins) has an affinity for GCC-like boxes in the promoter regions in Arabidopsis. In turn, DELLA protein reduces the promoter-binding activity of RAP2.3 [77].

We have also shown that the translation factors may hypothetically bind to polymorphic 5’ UTR. An analysis of the hypothetical RNA molecule secondary structure showed differences in the RNA conformation transcribed from different 5'-UTR variants, while the conformations of 5' -UTR-244 and 5' UTR-250 variants folded into more similar structures than 5' UTR-238 variant. Such differences can potentially significantly affect the efficiency of ribosome assembly and subsequent translation, since the 5’ UTR spatial form significantly affects the translation efficiency and can even inhibit it [78].

*GRF* can participate in the interaction with DELLA protein affecting nitrogen uptake and grain weight [10]. *TaGRF-2D* is localized with *Ppd-D1* at the same chromosome 2D [32, 34]. The combination of photoperiod insensitivity (*Ppd-D1a*) and dwarfing (*Rht-B1b*, *Rht-B1e*, and *Rht-D1b*) alleles are very important in wheat breeding especially in the Southern Europe [32, 65]. We searched for association between the polymorphisms in these loci and have revealed that the alleles are distributed independently in the studied bread wheat germplasms. We found that in the set of breeding lines the allelic state of *TaGRF-2D* is associated with thousand grain weight.

The 250 bp allele found in the majority of studied varieties was conditionally designated as the wild-type allele, whereas rarer 238 bp allele is supposed to be a mutant one. In *Ae*. *tauschii*, the 250 bp and 244 bp alleles were identified, the latter could result from a deletion of one hexanucleotide microsatellite GCAGCC. However, no *Ae*. *tauschii* accessions had an allele with a deletion of two GCAGCC junctions, i.e. the 12 bp deletion. It can be assumed that this rare deletion was locally found in individual *Ae*. *tauschii* populations involved in the formation of the bread wheat genome. Perhaps, the alternative version is also possible: this deletion was absent in *Ae*. *tauschii* and appeared as a result of domestication only in bread wheat, where it was fixed by selection. A sequential transition is also likely: the 244 bp allele with one deleted repeat unit from *Ae*. *tauschii* entered the genome of a bread wheat, after which a secondary deletion and a deletion of the second repeat unit occurred, which led to the formation of the 238 bp allele.

Two lineages of *Ae*. *tauschii*, L1 and L2, having practically non-overlapping areal are described [26]. Only *Ae*. *tauschii ssp*. *strangulata* (Eig) Tzvelev (eastern L2 lineage) is more likely to participate in bread wheat polyploidization; its geographical distribution overlaps the area of tetraploid wheat cultivation in Northern Iran at low elevations of Caspian Iran. But at the same time, it is 1D and 2D chromosomes carry the largest percentage of introgressions from the L1 lineages. For example, the *Lr22a* gene was introgressed into the bread wheat genome from lineage L1 of *Ae*. *tauschii* [79]. Although the botanical classification, according to the authors of the cited work, does not accurately reflect the division of *Ae*. *tauschii* into L1 and L2 lineages, it has been shown that most often *Ae*. *tauschii ssp*. *tauschii* and *ssp*. *anathera* subspecies belong to L1, and *Ae*. *tauschii* ssp. *strangulata*, ssp. *meyeri* and ssp. *typica* to L2 [26, 80–83]. If we combine the species in this way, it turns out that the 5' UTR-244 is more common than 5' UTR-250 (in 21 of 24 accessions) in L1, and the 5' UTR-244 and 5' UTR-250 alleles are found equally often in L2 (in 6 and 7 accessions, respectively). On the other hand, if we take into account only *Ae*. *tauschii* ssp. *strangulata* (as a D-genome donor) and *Ae*. *tauschii ssp*. *tauschii* (as a not D-genome donor) accessions, then it appears that the majority of *Ae*. *tauschii* ssp. *strangulata* species carry the 5' UTR-250 allele (more typical to bread wheat) while the majority of *Ae*. *tauschii ssp*. *tauschii* have 5' UTR-244. Interestingly, the majority of accessions of *Ae*. *tauschii* ssp. *strangulata* and *Ae*. *tauschii ssp*. *tauschii* that have 5' UTR-250 allele were collected from Iran or from Azerbaijan Districts that borders with Iran.

The presence of this deletion on 2D chromosome can be used to study the genetic diversity of bread wheat. Chromosome 2D is important for the breeding of bread wheat, as well as genome-substituted lines of durum wheat and triticale, since the *Ppd-D1* and *Rht8* genes are located on this chromosome. Perhaps, the allelic diversity at this locus is associated with dwarf phenotype and photoperiod insensitivity. We believe that the marker we developed can be used to assess the diversity of wheat and *Ae*. *tauschii* germplasm collections with respect to the *TaGRF-2D* locus and identify the most distant accessions of last species for further hybridization in order to develop more productive modern commercial cultivars of bread wheat and triticale.

## Conclusions

We have obtained the full-length *TaGRF-2D* gene sequence in bread wheat. The nucleotide sequences comparison between 18 varieties showed a high conservatism of the found sequences between each other in to the coding region and with the published TraesCS2D01G435200 sequence of Chinese Spring variety. We also did not reveal polymorphisms at the miR396 binding site, which theoretically could lead to differences in the expression product cleavage and thus lead to a different phenotype. At the same time, we showed a 12 bp polymorphism in the presence/absence of two GCAGCC microsatellite repeats in a 5’-untranslated region and revealed that studied varieties have either 5' UTR-238 or 5' UTR-250 allele. Since the 12 bp fragment was found in the majority of them, it is more likely that 5' UTR-250 is a wild-type allele, while 5' UTR-238 resulted from a 12 bp deletion.

## Supporting information

S1 Fig Agarose gel electrophoresis demonstrating polymorphism in amplification of CFD233 SSR-marker. Lanes: 1 –Saratovskaya 29, 2 –Novosibirskaya 67, 3 –Abigarib-3, 4 –Ibaa-95, 5 –Andry, 6 –Altigo, 7 –Pallada, 8 –Fisht.
(TIF)

S2 Fig Agarose gel electrophoresis demonstrating polymorphism in amplification of *Xgwm261*. Lanes: 1 –Ibaa-99 (165), 2 –Tomuz-3 (192), 3 –Abigarib-3 (165), 4 –Irak (165), 5 –Ibaa-95 (165), 6 –Andry (165), 7 –Romy (174), 8 –Sila (211).
(TIF)

S3 Fig Structure of *TaGRF-2D*. Non-coding part of chromosome, exons, introns, 5’- and 3’- UTRs, miRNA396 and polymorphic site in 5’ UTR are shown. Sites for PCR primers designed for the sequencing of overlapping regions are indicated by the arrows.
(TIF)

S4 Fig Agarose gel electrophoresis demonstrating the amplification of *TaGRF-2D* regions for the subsequent sequencing. The primers are shown as follows: a–GRF-2D-1F/1R; b–GRF-2D-2F/2R; c–GRF-2D-3F/3R; d–GRF-2D-4.1F/4.1R; e–GRF-2D-4.2F/4.2R; f–GRF-2D-5F/5R; g–GRF-2D-7F/7R.
(TIF)

S5 Fig Hypothetical binding sites of transcription factors in the 5’ UTR sequence of *TaGRF-2D*. (TIF)

S6 Fig Hypothetical secondary structures of the 5’ UTR RNA of *TaGRF-2D*. The structures transcribed from 5’ UTR of the following alleles are shown as follows: (a) 5’ UTR-238, (b) 5’ UTR-244, (c) 5’ UTR-250.
(TIF)

S1 Table Accession of bread wheat varieties and their allelic state of *TaGRF-2D* (GRF-2D-SSR fragment size). (DOCX)

S2 Table Accessions of *Ae*. *tauschii* and their allelic state of *TaGRF-2D* (GRF-2D-SSR fragment size). (DOCX)

S3 Table Primers and PCR conditions for the overlapping regions of *TaGRF-2D* designed for the sequencing and for the SRR marker GRF-2D-SSR designed for the indel identification in 5’ UTR of *TaGRF-2D*. (DOCX)

S4 Table The allelic state of *TaGRF-2D* (GRF-2D-SSR fragment size), *Rht-B1* and *Rht-D1* in the accessions of bread wheat varieties. (DOCX)

S5 Table The allelic state of *TaGRF-2D* (GRF-2D-SSR fragment size), *Rht-B1*, *Rht-D1*, and *Ppd-D1* and grain parameters in the studied bread wheat breeding lines. (DOCX)

S6 Table Analysis of variance (ANOVA) of the effects of *TaGRF-2D* and *Rht* on the grain parameters in the studied bread wheat breeding lines (for phenotypic data, see S5 Table). (DOCX)

S7 Table Predicted factors that hypothetically could bind to the found 5’ UTR sequence of *TaGRF-2D*. (DOCX)
