# Supplementary figures and images for: Allele mining of TaGRF-2D gene 5’-UTR in Triticum aestivum and Aegilops tauschii genotypes
Source: PLoS One. 2020 Apr 16;15(4):e0231704. doi: 10.1371/journal.pone.0231704 (PMC7162470; doi:10.1371/journal.pone.0231704)

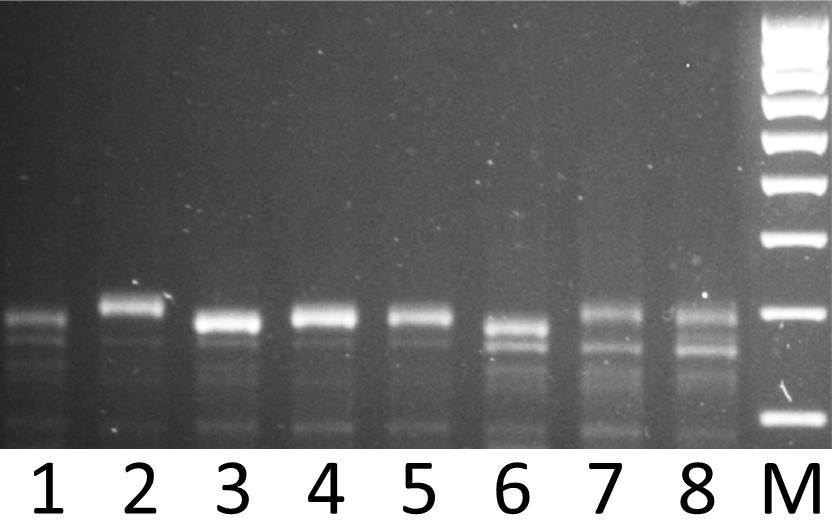

Supplement: S1 Fig — Lanes: 1 –Saratovskaya 29, 2 –Novosibirskaya 67, 3 –Abigarib-3, 4 –Ibaa-95, 5 –Andry, 6 –Altigo, 7 –Pallada, 8 –Fisht. (TIF) [file pone.0231704.s001.tif]

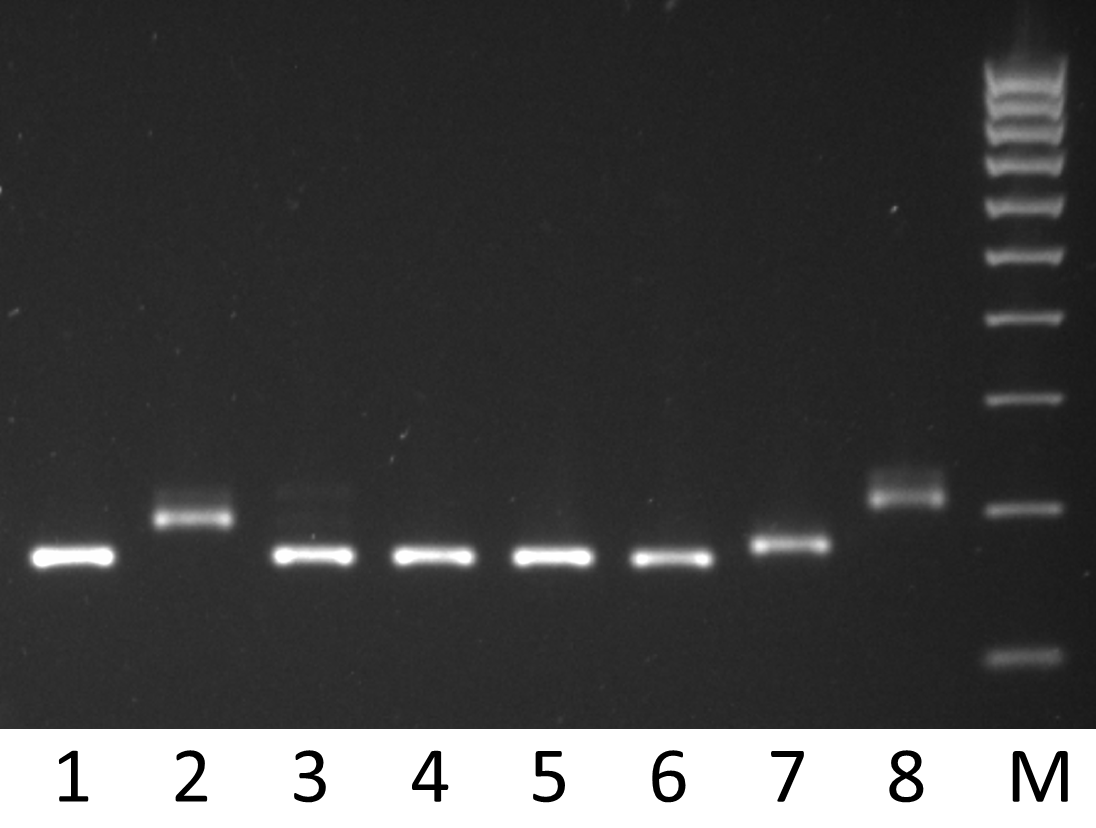

Supplement: S2 Fig — Lanes: 1 –Ibaa-99 (165), 2 –Tomuz-3 (192), 3 –Abigarib-3 (165), 4 –Irak (165), 5 –Ibaa-95 (165), 6 –Andry (165), 7 –Romy (174), 8 –Sila (211). (TIF) [file pone.0231704.s002.tif]

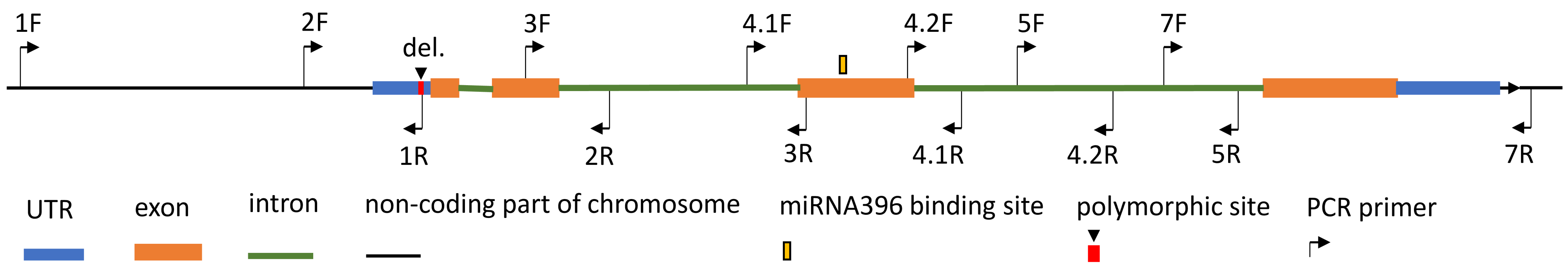

Supplement: S3 Fig — Non-coding part of chromosome, exons, introns, 5’- and 3’- UTRs, miRNA396 and polymorphic site in 5’ UTR are shown. Sites for PCR primers designed for the sequencing of overlapping regions are indicated by the arrows. (TIF) [file pone.0231704.s003.tif]

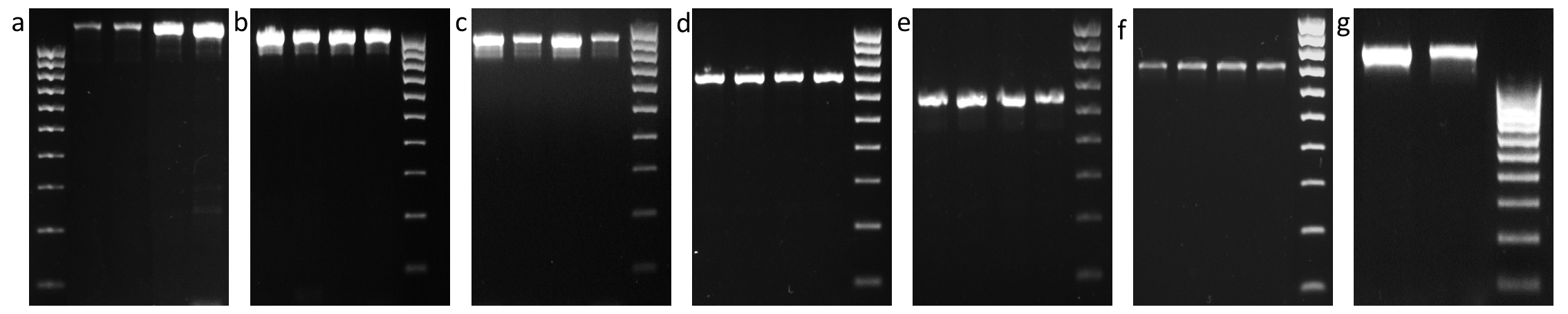

Supplement: S4 Fig — The primers are shown as follows: a–GRF-2D-1F/1R; b–GRF-2D-2F/2R; c–GRF-2D-3F/3R; d–GRF-2D-4.1F/4.1R; e–GRF-2D-4.2F/4.2R; f–GRF-2D-5F/5R; g–GRF-2D-7F/7R. (TIF) [file pone.0231704.s004.tif]

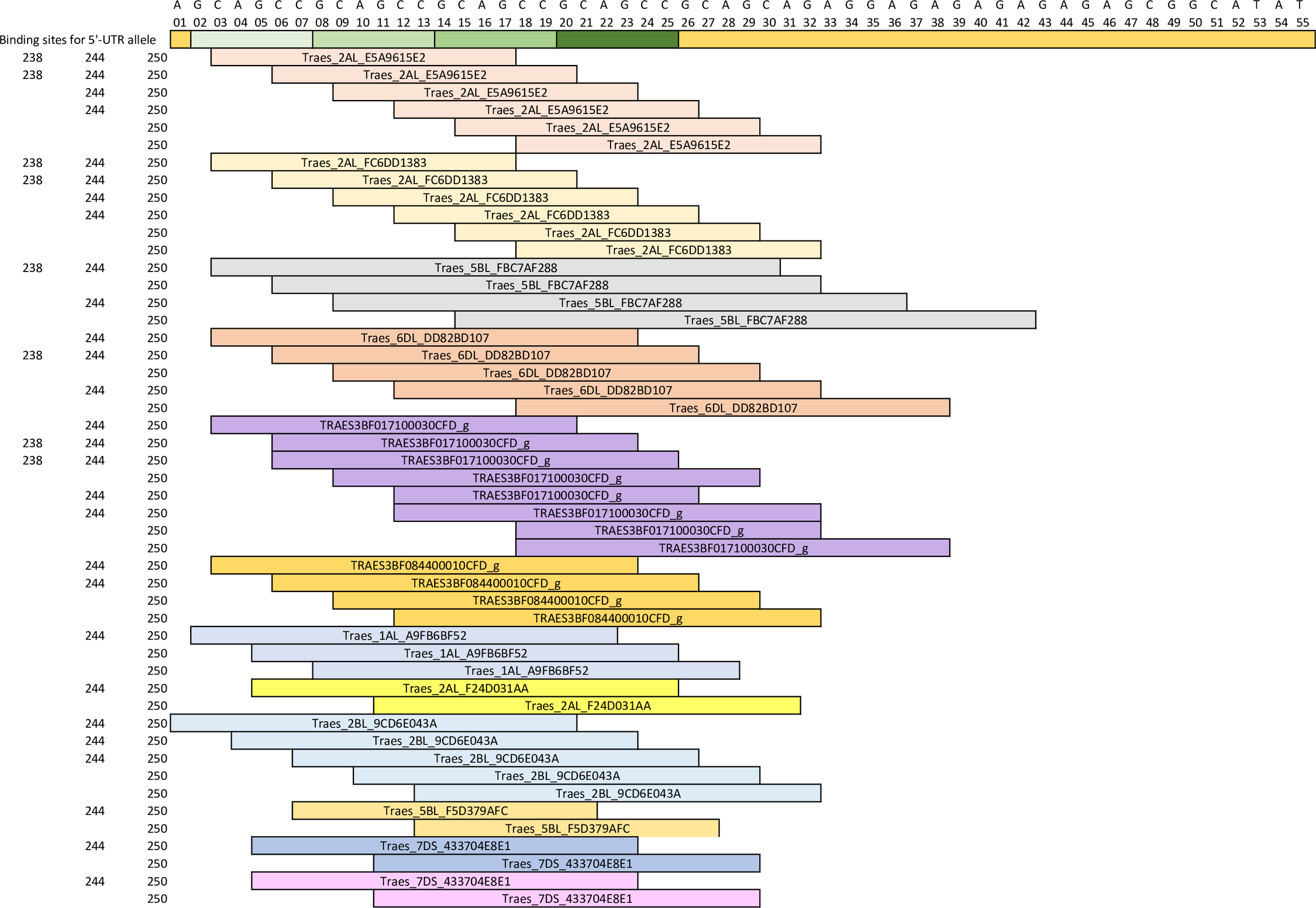

Supplement: S5 Fig — (TIF) [file pone.0231704.s005.tif]

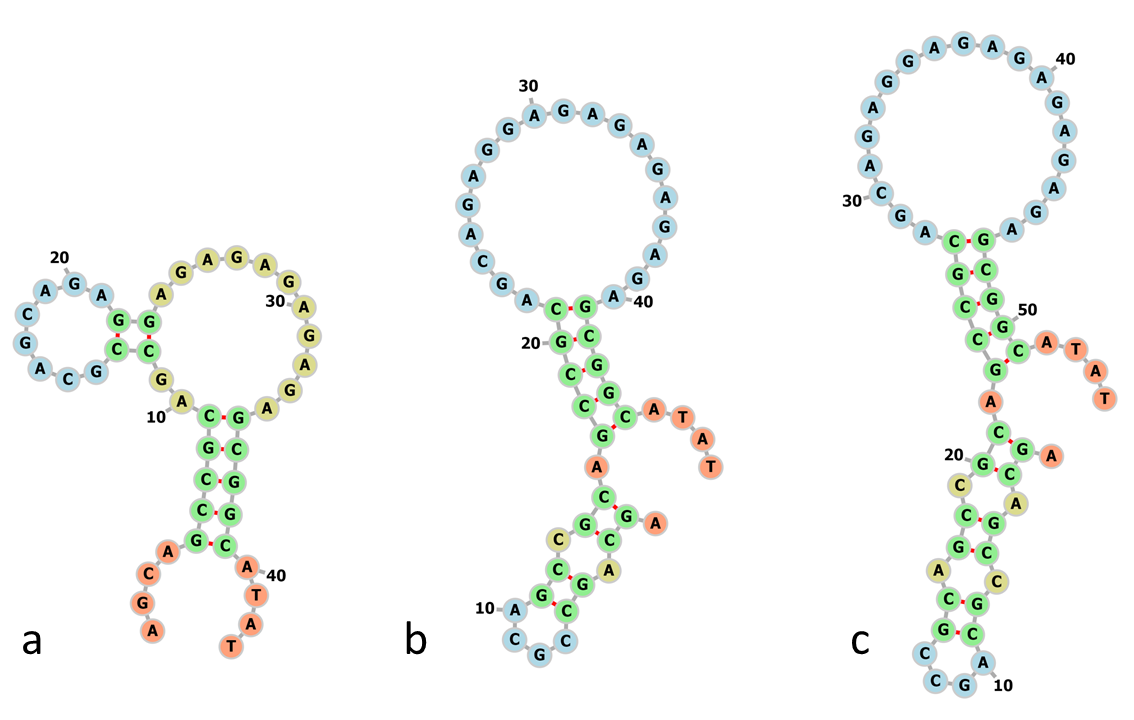

Supplement: S6 Fig — The structures transcribed from 5’ UTR of the following alleles are shown as follows: (a) 5’ UTR-238, (b) 5’ UTR-244, (c) 5’ UTR-250. (TIF) [file pone.0231704.s006.tif]
