# Supplementary material for: Allele mining of TaGRF-2D gene 5’-UTR in Triticum aestivum and Aegilops tauschii genotypes
Source: PLoS One. 2020 Apr 16;15(4):e0231704. doi: 10.1371/journal.pone.0231704 (PMC7162470; doi:10.1371/journal.pone.0231704)
Supplement: S1 Table — (DOCX) [file pone.0231704.s007.docx]

Allele mining of *TaGRF-2D* gene 5’-UTR

in *Triticum aestivum* and *Aegilops tauschii* genotypes.

Pavel Yu. Kroupin, Anastasiya G. Chernook, Mikhail S. Bazhenov, Gennady I. Karlov, Nikolay P. Goncharov, Nadezhda N. Chikida, and Mikhail G. Divashuk.

Supporting information

**S1 Table. Accession of bread wheat varieties and their allelic state of *TaGRF-2D* (GRF-2D-SSR fragment size).**

| **№** | **Variety of bread wheat** | **Origin** | **GRF-2D-SSR fragment size** |
| --- | --- | --- | --- |
|  | Ayvina | NCGL | 250 |
|  | Alekseich | NCGL | 238 |
|  | Al-Murooj | Iraq | 250 |
|  | Al-Rashid | Iraq | 250 |
|  | Altigo | France | 238 |
|  | Andry | Iraq | 250 |
|  | Anka | NCGL | 250 |
|  | Antonina | NCGL | 238 |
|  | Avrora | NCGL | 238 |
|  | Bash | NCGL | 250 |
|  | Abigarib-3 | Iraq | 250 |
|  | Bezostaya1 | NCGL | 250 |
|  | Bezostaya100 | NCGL | 238 |
|  | Brigada | NCGL | 238 |
|  | Dmitriy | NCGL | 250 |
|  | Doka | NCGL | 238 |
|  | Duplet | NCGL | 250 |
|  | Etnos | NCGL | 238 |
|  | Fatih | Iraq | 250 |
|  | Fisht | NCGL | 250 |
|  | Fortuna | NCGL | 238 |
|  | Gerda | NCGL | 250 |
|  | Graf | NCGL | 238 |
|  | Grom | NCGL | 250 |
|  | Gurt | NCGL | 250 |
|  | Ibaa-95 | Iraq | 250 |
|  | Ibaa-99 | Iraq | 250 |
|  | Iraq | Iraq | 250 |
|  | Jiva | NCGL | 238 |
|  | Kalym | NCGL | 250 |
|  | Karavan | NCGL | 250 |
|  | Kavalerka | NCGL | 250 |
|  | Kavkaz | NCGL | 250 |
|  | Knyazhna | NCGL | 250 |
|  | Korotyshka | NCGL | 250 |
|  | Krasnodarskaya99 | NCGL | 250 |
|  | Krasota | NCGL | 238 |
|  | Kroshka | NCGL | 238 |
|  | Kuren' | NCGL | 250 |
|  | Lebed' | NCGL | 250 |
|  | Markiz | NCGL | 250 |
|  | Morozko | NCGL | 238 |
|  | Nota | NCGL | 250 |
|  | Novosibirskaya 32 | ICG | 250 |
|  | Novosibirskaya 67 | ICG | 250 |
|  | Pallada | NCGL | 250 |
|  | Pamyat' | NCGL | 238 |
|  | Pervitsa | NCGL | 250 |
|  | Pobeda50 | NCGL | 250 |
|  | Polovchanka | NCGL | 238 |
|  | Proton | NCGL | 250 |
|  | Romy | Iraq | 250 |
|  | Saratovskaya 29 | ARI | 250 |
|  | Sham-6 | Iraq | 250 |
|  | Shkola | NCGL | 250 |
|  | Sila | NCGL | 250 |
|  | Skifyanka | NCGL | 250 |
|  | Soberbash | NCGL | 238 |
|  | Stan | NCGL | 250 |
|  | Starshina | NCGL | 238 |
|  | Step' | NCGL | 238 |
|  | Svarog | NCGL | 238 |
|  | Tanya | NCGL | 250 |
|  | Timiryazevka150 | NCGL | 238 |
|  | Tomuz-3 | Iraq | 250 |
|  | Ul'tra11 | NCGL | 238 |
|  | Ul'tra9 | NCGL | 238 |
|  | Urup | NCGL | 250 |
|  | Utrish | NCGL | 238 |
|  | Vanya | NCGL | 250 |
|  | Vassa | NCGL | 250 |
|  | Veha | NCGL | 250 |
|  | Velena | NCGL | 250 |
|  | Vid | NCGL | 250 |
|  | Videya | NCGL | 250 |
|  | Viza | NCGL | 250 |
|  | Vostorg | NCGL | 250 |
|  | Yubileinaya 100 | NCGL | 238 |
|  | Zimtra | NCGL | 238 |

NCGL, provided by Department of Breeding and Seed Production of Wheat and Triticale, National Center of Grain named after P.P. Lukyanenko (Krasnodar, Russia)

ICG, provided by provided by Federal Research Center Institute of Cytology and Genetics, Siberian Branch of Russian Academy of Sciences (Novosibirsk, Russia)

ARI, provided by Agricultural Research Institute of South-East Region (Saratov, Russia)

Iraq, provided by Dr. Oleg G. Semenov (Department of Technosphere Safety, Agrarian-Technological Institute, RUDN University, Moscow)
