# Supplementary material for: Allele mining of TaGRF-2D gene 5’-UTR in Triticum aestivum and Aegilops tauschii genotypes
Source: PLoS One. 2020 Apr 16;15(4):e0231704. doi: 10.1371/journal.pone.0231704 (PMC7162470; doi:10.1371/journal.pone.0231704)
Supplement: S2 Table — (DOCX) [file pone.0231704.s008.docx]

Allele mining of *TaGRF-2D* gene 5’-UTR

in *Triticum aestivum* and *Aegilops tauschii* genotypes.

Pavel Yu. Kroupin, Anastasiya G. Chernook, Mikhail S. Bazhenov, Gennady I. Karlov, Nikolay P. Goncharov, Nadezhda N. Chikida, and Mikhail G. Divashuk.

Supporting information

**S2 Table. Accessions of *Ae. tauschii* and their allelic state of *TaGRF-2D* (GRF-2D-SSR fragment size).**

| **№** | **Accession of *Aegilops tauschii*** | **Origin** | **GRF-2D-SSR fragment size** |
| --- | --- | --- | --- |
|  | *Ae. tauschii* ssp. *strangulata* К-4056 | Iran | 250 |
|  | *Ae. tauschii* ssp. *strangulata* К-108 | Azerbaijan (Massalin District) | 250 |
|  | *Ae. tauschii* ssp. *tauschii* К-4564 | Syria | 244 |
|  | *Ae. tauschii* ssp. *strangulata* К-112 | Azerbaijan (Jalilabad District) | 250 |
|  | *Ae. tauschii* ssp. *tauschii* К-1216 | Georgia | 244 |
|  | *Ae. tauschii* ssp. *strangulata* К-1662 | Iran | 250 |
|  | *Ae. tauschii* ssp. *strangulata* К-113 | Azerbaijan | 244 |
|  | *Ae. tauschii* ssp. *tauschii* К-2271 | Armenia (Kotayk Province) | 250 |
|  | *Ae. tauschii* ssp. *tauschii* К-396 | Uzbekistan | 244 |
|  | *Ae. tauschii* ssp. *tauschii* К-527 | Armenia | 244 |
|  | *Ae. tauschii* ssp. *tauschii* К-1770 | Russia (Dagestan) | 244 |
|  | *Ae. tauschii* ssp. *tauschii* К-4049 | Iran | 244 |
|  | *Ae. tauschii* ssp. *tauschii* К-1657 | Palestine | 244 |
|  | *Ae. tauschii* ssp. *tauschii* К-1099 | Azerbaijan (Lerik District) | 250 |
|  | *Ae. tauschii* ssp. *tauschii* К-1112 | Azerbaijan | 244 |
|  | *Ae. tauschii* ssp. *tauschii* К-1723 | Azerbaijan | 244 |
|  | *Ae. tauschii* ssp. *tauschii* К-994 | Afghanistan | 244 |
|  | *Ae. tauschii* var. *meyeri* К-608 | Georgia | 244 |
|  | *Ae. tauschii* *ssp. typica* К-3187 | Armenia | 250 |
|  | *Ae. tauschii* ssp. *tauschii* К-394 | Uzbekistan | 244 |
|  | *Ae. tauschii* ssp. *tauschii* var. *typica* К-428 | Turkmenistan | 244 |
|  | *Ae. tauschii* ssp. *strangulata* С 21-5129 (Praga-Ruzine) | Azerbaijan | 244 |
|  | *Ae. tauschii* ssp. *tauschii* С 21-5144 | Turkmenistan | 244 |
|  | *Ae. tauschii* ssp. *strangulata* С 21-5118 | Iran | 250 |
|  | *Ae. tauschii* ssp. *strangulata* С 21-5127 | Azerbaijan | 244 |
|  | *Ae. tauschii* ssp. *tauschii* С 21-5130 | Azerbaijan | 244 |
|  | *Ae. tauschii* ssp. *tauschii* С 21-4030 | Unknown | 244 |
|  | *Ae. tauschii* ssp. *tauschii* С 21-5143 | Pakistan | 244 |
|  | *Ae. tauschii* ssp. *tauschii* К-1336 | Kazakhstan | 244 |
|  | *Ae. tauschii* ssp. *tauschii* var. *typica* КU-2001 | Pakistan | 244 |
|  | *Ae. tauschii* ssp. *tauschii* var. *anathera* КU-2003 | Pakistan | 244 |
|  | *Ae. tauschii* ssp. *strangulata* KU-2074 | Iran | 250 |
|  | *Ae. tauschii* ssp. *tauschii* K-896 | Afghanistan | 244 |
|  | *Ae. tauschii* ssp. *tauschii* К-865 | Unknown | 244 |
|  | *Ae. tauschii* ssp. *tauschii* KT 120-13 | China | 244 |
|  | *Ae. tauschii* ssp. *tauschii* KT 120-10 | China | 250 |
|  | *Ae. tauschii* ssp. *tauschii* Ae - 111040 | Syria | 244 |

The *Ae. tauschii* accessions partially were provided by the Federal Research Center Vavilov All-Russian Institute of Plant Genetic Resources (VIR), Saint-Petersburg, Russia (“K –“); Czech Institute of Plant Industry, Praga-Ruzine, Czech Republic (“С 21 -“); Kyoto University, Kyoto, Japan (“KU –“); Institute Biology of Kihara Foundation, Yokohama, Japan (“KT –“); ICARDA, Aleppo, Syria (“Ae –“).
