# Supplementary material for: Allele mining of TaGRF-2D gene 5’-UTR in Triticum aestivum and Aegilops tauschii genotypes
Source: PLoS One. 2020 Apr 16;15(4):e0231704. doi: 10.1371/journal.pone.0231704 (PMC7162470; doi:10.1371/journal.pone.0231704)
Supplement: S3 Table — (DOCX) [file pone.0231704.s009.docx]

Allele mining of *TaGRF-2D* gene 5’-UTR

in *Triticum aestivum* and *Aegilops tauschii* genotypes.

Pavel Yu. Kroupin, Anastasiya G. Chernook, Mikhail S. Bazhenov, Gennady I. Karlov, Nikolay P. Goncharov, Nadezhda N. Chikida, and Mikhail G. Divashuk.

Supporting information

**Table S3. Primers and PCR conditions for the overlapping regions of *TaGRF-2D* designed for the sequencing and for the SRR marker GRF-2D-SSR designed for the indel identification in 5’ UTR of *TaGRF-2D*.**

| **Title** | **Primer sequence** | **PCR mixture** | **Annealing temperature, °C** | **Expected size, bp** |
| --- | --- | --- | --- | --- |
| GRF-2D-1 | F: 5' CCC GTC ATC AAG TGA CAA CTC AG 3'  R: 5' GCC GTG CTT TTG CCT TTT ATT AGT 3' | 1x LR buffer (pH=9,3), 2,5 mM MgCl_2_, 0,2 mM of each dNTP, 2 µM of each primer, 0,04 U/µl LR Plus polymerase, 0,02 U/µl Taq polymerase, 4 ng/µl DNA template | 61 | 1223 |
| GRF-2D-2 | F: 5' CTC CGG TGG TGA ACT TTG TTT TA 3'  R: 5' TAC ACC TGA ATC TCT CGA TCC CA 3' | 1x LR buffer (pH=9,3), 1,5 mM MgCl_2_, 0,2 mM of each dNTP, 2 µM of each primer, 0,04 U/µl LR Plus polymerase, 0,02 U/µl Taq polymerase, 4 ng/µl DNA template | 60 | 1009 |
| GRF-2D-3 | F: 5' GGC GCT CAT CTA CAA GTA CCT C 3'  R: 5' CGA CGG GTA CAA ATC AAA TCA GG 3' | 1x LR buffer (pH=9,3), 1,5 mM MgCl_2_, 0,2 mM of each dNTP, 2 µM of each primer, 0,04 U/µl LR Plus polymerase, 0,02 U/µl Taq polymerase, 4 ng/µl DNA template | 60 | 903 |
| GRF-2D-4.1 | F: 5' ACA GAC AGA TAG ATC CAG GAC GA 3'  R: 5' CAC ATG TTA TGG GCC ACT TCA TC 3' | 1x LR buffer (pH=9,3), 1,5 mM MgCl_2_, 0,2 mM of each dNTP, 2 µM of each primer, 0,04 U/µl LR Plus polymerase, 0,02 U/µl Taq polymerase, 4 ng/µl DNA template, 10% v/v DMSO | 60 | 676 |
| GRF-2D-4.2 | F: 5' CGG AAA CAA GGA TTT CAG GTG AT 3'  R: 5' AGT GAC TCG TAC GGA GCA AAA TA 3' | 1x LR buffer (pH=9,3), 1,5 mM MgCl_2_, 0,2 mM of each dNTP, 2 µM of each primer, 0,04 U/µl LR Plus polymerase, 0,02 U/µl Taq polymerase, 4 ng/µl DNA template, 10% v/v DMSO | 60 | 643 |
| GRF-2D-5 | F: 5' TCT GCC TGC AAG AGA TGA AAC T 3'  R: 5' AGA CAA AAC AAA AGG GAC ATA GC 3'C | 1x LR buffer (pH=9,3), 1,5 mM MgCl_2_, 0,2 mM of each dNTP, 2 µM of each primer, 0,04 U/µl LR Plus polymerase, 0,02 U/µl Taq polymerase, 4 ng/µl DNA template | 60 | 697 |
| GRF-2D-7 | F: 5' CCT GTC ATT GTA GTA GGG TTG CT 3'  R: 5' CCA TCA TCA AAA GGA GGA TGC TG 3' | 1x LR buffer (pH=9,3), 1,5 mM MgCl_2_, 0,2 mM of each dNTP, 2 µM of each primer, 0,04 U/µl LR Plus polymerase, 0,02 U/µl Taq polymerase, 4 ng/µl DNA template, 4% v/v DMSO | 60 | 1342 |
| GRF-2D-SSR | F: 5' CAT CCC ACT GCC CCC ATC GT 3'  R: FAM-5' GAG GCA AAG GGC ATC GCC ATA 3' | 1x LR buffer (pH=9,3), 1,5 mM MgCl_2_, 0,2 mM of each dNTP, 2 µM of each primer, 0,04 U/µl LR Plus polymerase, 0,02 U/µl Taq polymerase, 4 ng/µl DNA template | 63 | 246 |
