# Supplementary material for: Allele mining of TaGRF-2D gene 5’-UTR in Triticum aestivum and Aegilops tauschii genotypes
Source: PLoS One. 2020 Apr 16;15(4):e0231704. doi: 10.1371/journal.pone.0231704 (PMC7162470; doi:10.1371/journal.pone.0231704)
Supplement: S4 Table — (DOCX) [file pone.0231704.s010.docx]

Allele mining of *TaGRF-2D* gene 5’-UTR

in *Triticum aestivum* and *Aegilops tauschii* genotypes.

Pavel Yu. Kroupin, Anastasiya G. Chernook, Mikhail S. Bazhenov, Gennady I. Karlov, Nikolay P. Goncharov, Nadezhda N. Chikida, and Mikhail G. Divashuk.

Supporting information

**S4 Table. The allelic state of** ***TaGRF-2D* (GRF-2D-SSR fragment size), *Rht-B1* and *Rht-D1* in the accessions of bread wheat varieties.**

| **№** | **Variety of bread wheat** | **GRF-2D-SSR fragment size** | ***Rht-B1*** | ***Rht-D1*** |
| --- | --- | --- | --- | --- |
|  | Alekseich | 238 | *Rht-B1b* | *Rht-D1a* |
|  | Al-Murooj | 250 | *Rht-B1b* | *Rht-D1a* |
|  | Altigo | 238 | *Rht-B1a* | *Rht-D1b* |
|  | Doka | 238 | *Rht-B1e* | *Rht-D1a* |
|  | Fisht | 250 | *Rht-B1e* | *Rht-D1a* |
|  | Grom | 250 | *Rht-B1e* | *Rht-D1a* |
|  | Iraq | 250 | *Rht-B1b* | *Rht-D1a* |
|  | Novosibirskaya 67 | 250 | *Rht-B1a* | *Rht-D1a* |
|  | Proton | 250 | *Rht-B1a* | *Rht-D1b* |
|  | Romy | 250 | *Rht-B1b* | *Rht-D1a* |
|  | Saratovskaya 29 | 250 | *Rht-B1a* | *Rht-D1a* |
|  | Sila | 250 | *Rht-B1b* | *Rht-D1a* |
|  | Soberbash | 238 | *Rht-B1b* | *Rht-D1a* |
|  | Stan | 250 | *Rht-B1b* | *Rht-D1a* |
|  | Tomuz-3 | 250 | *Rht-B1b* | *Rht-D1a* |
|  | Vassa | 250 | *Rht-B1b* | *Rht-D1a* |
|  | Velena | 250 | *Rht-B1e* | *Rht-D1a* |
|  | Vid | 250 | *Rht-B1b* | *Rht-D1a* |
