# Supplementary material for: Allele mining of TaGRF-2D gene 5’-UTR in Triticum aestivum and Aegilops tauschii genotypes
Source: PLoS One. 2020 Apr 16;15(4):e0231704. doi: 10.1371/journal.pone.0231704 (PMC7162470; doi:10.1371/journal.pone.0231704)
Supplement: S5 Table — (DOCX) [file pone.0231704.s011.docx]

Allele mining of *TaGRF-2D* gene 5’-UTR

in *Triticum aestivum* and *Aegilops tauschii* genotypes.

Pavel Yu. Kroupin, Anastasiya G. Chernook, Mikhail S. Bazhenov, Gennady I. Karlov, Nikolay P. Goncharov, Nadezhda N. Chikida, and Mikhail G. Divashuk.

Supporting information

**S5 Table. The allelic state of *TaGRF-2D* (GRF-2D-SSR fragment size), *Rht-B1*, *Rht-D1*, and *Ppd-D1* and grain parameters in the studied bread wheat breeding lines.**

| **№** | **Breeding line** | **GRF-2D-SSR fragment size** | ***Rht-B1*** | ***Rht-D1*** | ***Ppd-D1*** | **Grain length, cm** | **Grain width, cm** | **Thousand grain weight, g** |
| --- | --- | --- | --- | --- | --- | --- | --- | --- |
|  | 3h | 250 | *Rht-B1a* | *Rht-D1b* | *Ppd-D1a* | 4.7 | 2.1 | 33.7 |
|  | 4h | 250 | *Rht-B1a* | *Rht-D1a* | *Ppd-D1a* | 4.9 | 2.1 | 35.9 |
|  | 5h | 250 | *Rht-B1b* | *Rht-D1a* | *Ppd-D1b* | 4.5 | 2.1 | 32.4 |
|  | 6h | 238 | *Rht-B1a* | *Rht-D1a* | *Ppd-D1b* | 5.0 | 2.1 | 35.3 |
|  | 20h | 250 | *Rht-B1b* | *Rht-D1a* | *Ppd-D1b* | 4.9 | 2.1 | 32.5 |
|  | 24h | 250 | *Rht-B1b* | *Rht-D1b* | *Ppd-D1b* | 4.3 | 2.2 | 33.8 |
|  | 25h | 238 | *Rht-B1b* | *Rht-D1a* | *Ppd-D1a* | 5.1 | 2.4 | 31.7 |
|  | 27h | 238 | *Rht-B1a* | *Rht-D1a* | *Ppd-D1b* | 5.0 | 2.2 | 38.5 |
|  | 32h | 250 | *Rht-B1e* | *Rht-D1a* | *Ppd-D1b* | 5.4 | 2.4 | 34.2 |
|  | 33h | 250 | *Rht-B1b* | *Rht-D1a* | *Ppd-D1a* | 4.7 | 2.1 | 34.4 |
|  | 39h | 238 | *Rht-B1a* | *Rht-D1a* | *Ppd-D1b* | 4.9 | 2.2 | 38.0 |
|  | 50h | 238 | *Rht-B1b* | *Rht-D1a* | *Ppd-D1a* | 5.4 | 2.4 | 32.5 |
|  | 52h | 250 | *Rht-B1b* | *Rht-D1a* | *Ppd-D1a* | 5.0 | 2.4 | 31.7 |
|  | 56h | 238 | *Rht-B1b* | *Rht-D1a* | *Ppd-D1b* | 4.9 | 2.2 | 35.7 |
|  | 76h | 238 | *Rht-B1b* | *Rht-D1a* | *Ppd-D1a* | 5.5 | 2.3 | 32.5 |
|  | 99h | 238 | *Rht-B1b* | *Rht-D1a* | *Ppd-D1a* | 4.9 | 2.1 | 36.2 |
|  | 104h | 238 | *Rht-B1b* | *Rht-D1a* | *Ppd-D1b* | 5.3 | 2.6 | 38.9 |
|  | 109h | 238 | *Rht-B1b* | *Rht-D1a* | *Ppd-D1a* | 5.5 | 2.5 | 38.0 |
|  | 110h | 250 | *Rht-B1a* | *Rht-D1b* | *Ppd-D1a* | 5.2 | 2.4 | 34.0 |
|  | 138h | 238 | *Rht-B1b* | *Rht-D1a* | *Ppd-D1a* | 5.8 | 2.5 | 37.4 |
