# Supplementary material for: Allele mining of TaGRF-2D gene 5’-UTR in Triticum aestivum and Aegilops tauschii genotypes
Source: PLoS One. 2020 Apr 16;15(4):e0231704. doi: 10.1371/journal.pone.0231704 (PMC7162470; doi:10.1371/journal.pone.0231704)
Supplement: S6 Table — (DOCX) [file pone.0231704.s012.docx]

Allele mining of *TaGRF-2D* gene 5’-UTR

in *Triticum aestivum* and *Aegilops tauschii* genotypes.

Pavel Yu. Kroupin, Anastasiya G. Chernook, Mikhail S. Bazhenov, Gennady I. Karlov, Nikolay P. Goncharov, Nadezhda N. Chikida, and Mikhail G. Divashuk.

Supporting information

**S6 Table. Analysis of variance (ANOVA) of the effects of *TaGRF-2D* and *Rht* on the grain parameters in the studied bread wheat breeding lines (for phenotypic data, see Table S5).**

| ***TaGRF-2D* allelic state** | **Mean GL** | **Standard Error** | **Number of breeding lines** | **ANOVA of the effect of the *TaGRF-2D* allelic state on grain length** | | | | | | | | |
| --- | --- | --- | --- | --- | --- | --- | --- | --- | --- | --- | --- | --- |
| 5’ UTR-238 | 5.2a* | 0.09 | 11 | **Variance** | **Sum of Squares** | | **Degree of Freedom** | | **Mean square** | | **F** | **p** |
|  |  |  |  | Intercept | 500.3 | | 1 | | 500.3 | | 4812.5 | 0.000 |
| 5’ UTR-250 | 4.8b | 0.11 | 9 | *TaGRF-2D* | 0.7 | | 1 | | 0.7 | | 6.3 | 0.022 |
|  |  |  |  | Error | 1.9 | | 18 | | 0.1 | |  |  |
| ***TaGRF-2D* allelic state** | **Mean GW** | **Standard Error** | **Number of breeding lines** | **ANOVA of the effect of the *TaGRF-2D* allelic state on grain width** | | | | | | | | |
| 5’ UTR-238 | 2.3a | 0.05 | 11 | **Variance** | **Sum of Squares** | | **Degree of Freedom** | | **Mean square** | | **F** | **p** |
|  |  |  |  | Intercept | 101.5 | | 1 | | 101.5 | | 3928.7 | 0.000 |
| 5’ UTR-250 | 2.2a | 0.05 | 9 | *TaGRF-2D* | 0.1 | | 1 | | 0.1 | | 2.2 | 0.156 |
|  |  |  |  | Error | 0.5 | | 18 | | 0.0 | |  |  |
| ***TaGRF-2D* allelic state** | **Mean TGW** | **Standard Error** | **Number of breeding lines** | **ANOVA of the effect of the *TaGRF-2D* allelic state on TGW** | | | | | | | | |
| 5’ UTR-238 | 35.9a | 0.79 | 11 | **Variance** | | **Sum of Squares** | | **Degree of Freedom** | | **Mean square** | **F** | **p** |
|  |  |  |  | Intercept | | 23912.5 | | 1 | | 23912.5 | 5324.9 | 0.000 |
| 5’ UTR-250 | 33.6b | 0.42 | 9 | *TaGRF-2D* | | 25.3 | | 1 | | 25.3 | 5.6 | 0.029 |
|  |  |  |  | Error | | 80.8 | | 18 | | 4.5 |  |  |
| ***Rht* allelic state** | **Mean TGW** | **Standard Error** | **Number of breeding lines** | **ANOVA of the effect of the *Rht* allelic state on TGW** | | | | | | | | |
| *Rht-B1a,*  *Rht-D1a* | 36.9a | 0.78 | 4 | **Variance** | | **Sum of Squares** | | **Degree of Freedom** | | **Mean square** | **F** | **p** |
|  |  |  |  | Intercept | | 16256.4 | | 1 | | 16256.40 | 3447.1 | 0.000 |
| *Rht-B1b,*  *Rht-B1e, Rht-D1b* | 34.4b | 0.57 | 16 | *Rht* | | 21.2 | | 1 | | 21.22 | 4.5 | 0.048 |
|  |  |  |  | Error | | 84.9 | | 18 | | 4.72 |  |  |

* - the mean values that do not differ significantly (p < 0.05) are shown with the same letters

GL, grain length; GW, grain width, TGW, thousand grain weight
