# Supplementary material for: Allele mining of TaGRF-2D gene 5’-UTR in Triticum aestivum and Aegilops tauschii genotypes
Source: PLoS One. 2020 Apr 16;15(4):e0231704. doi: 10.1371/journal.pone.0231704 (PMC7162470; doi:10.1371/journal.pone.0231704)
Supplement: S7 Table — (DOCX) [file pone.0231704.s013.docx]

Allele mining of *TaGRF-2D* gene 5’-UTR

in *Triticum aestivum* and *Aegilops tauschii* genotypes.

Pavel Yu. Kroupin, Anastasiya G. Chernook, Mikhail S. Bazhenov, Gennady I. Karlov, Nikolay P. Goncharov, Nadezhda N. Chikida, and Mikhail G. Divashuk.

Supporting information

**S5 Table. Predicted factors that hypothetically could bind to the found 5’ UTR sequence of *TaGRF-2D.***

| **ID** | **Predicted translation factor** |
| --- | --- |
| U0001 | HSL3- Histone 3'UTR stem-loop structure (HSL3) |
| U0002 | IRE - Iron Responsive Element (IRE) |
| U0003 | SECIS1- Selenocysteine Insertion Sequence - type 1 (SECIS1) |
| U0004 | SECIS2- Selenocysteine Insertion Sequence - type 2 (SECIS2) |
| U0005 | APP_SCE - Amyloid precursor protein mRNA stability control element (APP_SCE) |
| U0006 | CPE - Cytoplasmic polyadenylation element (CPE) |
| U0007 | TGE - TGE translational regulation element (TGE) |
| U0008 | NANOS_TCE - Nanos translation control element (NANOS_TCE) |
| U0009 | 15-LOX-DICE - 15-Lipoxygenase Differentiation Control Element (15-LOX-DICE) |
| U0010 | ARE2- AU-rich class-2 Element (ARE2) |
| U0011 | TOP - Terminal Oligopyrimidine Tract (TOP) |
| U0012 | GLUT1 - Glusose transporter type-1 3'UTR cis-acting element (GLUT1) |
| U0013 | TNF - Tumor necrosis factor alpha 3'UTR cis-acting element (TNF) |
| U0014 | VIM3- Vimentin 3'UTR cis-acting element (VIM3) |
| U0015 | IRES- Internal Ribosome Entry Site (IRES) |
| U0016 | SXL_BS- SXL binding site |
| U0017 | UNR-bs- UNR binding site |
| U0018 | RPMS12_TCE- Ribosomal S12 mitochondrial protein 5'UTR translation control element (RPMS12_TCE) |
| U0019 | BRE - Bruno 3'UTR responsive element (BRE) |
| U0020 | ADH_DRE - Alcohol dehydrogenase 3'UTR downregulation control element (ADH_DRE) |
| U0021 | BYDV_TE - Barley yellow dwarf virus translation control element (BYDV_TE) |
| U0022 | PRONEURAL-BOX - Proneural Box (PB) |
| U0023 | K-BOX - K-Box (KB) |
| U0024 | BRD-BOX - Brd-Box (Brd) |
| U0025 | GY-BOX- GY-Box (GY) |
| U0026 | AR_CURE - Androgen receptor CU-rich element (AR_CURE) |
| U0027 | G3A - Elastin G3A 3'UTR stability motif (G3A) |
| U0028 | INS_SCE - Insulin 3'UTR stability element (INS_SCE) |
| U0029 | ACTIN_ZIP3- Beta-actin 3'UTR zipcode (ACTIN_ZIP3) |
| U0030 | GAP-43- Gap-43 Stabilization Element (GAP-43) |
| U0031 | CNDLE - CaMKII/Ng dendritic localization element (CNDLE) |
| U0033 | uORF- Upstream Open Reading Frame (uORF) |
| U0032 | AG-CRSD - alpha-globin 3'UTR C-rich stability determinant (AG-CRSD) |
| U0034 | GAIT- Gamma interferon activated inhibitor of translation (GAIT element) |
| U0036 | HLE - Drosophila hairy mRNA localization element (HLE) |
| U0037 | MBP-A2RE11- Myelin Basic Protein Localization Element (MBP-A2RE11) |
| U0038 | Protamine-YRS - Protamine P1 3'UTR Y-Box recognition site (Protamine-YRS) |
| U0039 | G-CSF_SLDE- Granulocyte colony-stimulating factor stem-loop destabilizing element (G-CSF_SLDE) |
| U0040 | Ren_SRE - Renin stability regulatory element (Ren_SRE) |
| U0041 | PTH1- PTH 3'UTR proximal cis-acting instability element (PTH1) |
| U0042 | PTH2- PTH 3'UTR distal cis-acting instability element (PTH2) |
| U0043 | PAS - Polyadenylation Signal (PAS) |
| U0044 | PABP_ARS- PABP mRNA autoregulatoy repression sequence |
| U0045 | ApoB- ApoB 5'UTR cis-acting regulatory element |
| U0046 | TPP_riboswitch- Thiamin pyrophosphate riboswitch (TPP_riboswitch) |
